# Recurrent Hydatidiform Moles: A Clinical Challenge—A Case Report and an Update on Management and Therapeutical Strategies

**DOI:** 10.1155/2023/3752274

**Published:** 2023-06-20

**Authors:** S. Riccio, F. Galanti, M. Scudo, L. Di Troia, M. G. Ferrillo, F. Manzara, P. Ianiri, F. A. Battaglia

**Affiliations:** Obstetrics and Gynecology Unit, Santa Maria Goretti Hospital, Via Canova, Latina 04100, Italy

## Abstract

Hydatidiform mole, complete or partial (CHM/PHM), is the most common type of gestational trophoblastic disease (GTD), which is characterized by excessive trophoblastic proliferation and abnormal embryonic development. Some patients present with sporadic or familiar recurrent hydatidiform moles (RHMs), which are characterized by two or more episodes of the disease. A healthy 36-year-old woman was admitted to the Obstetrics and Gynecology Unit of Santa Maria Goretti Hospital, Latina, because of RHMs at 6 weeks of amenorrhea, with an obstetrical anamnesis of RHMs. We performed uterine dilatation and curettage with suction evacuation. The histological examination confirmed the diagnosis of PHM. The clinical follow-up was conducted according to recent guidelines on the diagnosis and management of GTD. After the return to the baseline values of the beta-human chorionic gonadotropin hormone, a combined oral contraceptive therapy was proposed, and the patient was invited to undergo in vitro fertilization (IVF) techniques, specifically oocyte donation, to reduce the possibility of similar future cases of RHMs. Although some etiopathogenetic mechanisms involved in RHMs are still unknown, all patients of childbearing age who are affected by this syndrome should be properly treated and directed towards a correct clinical path as IVF, to have a successful and safe pregnancy.

## 1. Introduction

Gestational trophoblastic disease (GTD) is a spectrum of cellular proliferations arising from the placental villous trophoblast including four main clinical forms: complete hydatidiform mole (CHM), partial hydatidiform mole (PHM), invasive mole, choriocarcinoma, and placental site trophoblastic tumour (PSTT), and epithelioid trophoblastic tumor (ETT) [1]. The latter four malignancies are collectively defined as gestational trophoblastic neoplasms (GTN).

The GTD spectrum has recently been expanded to include atypical placental site nodules, which may coexist and develop into PSTT/ETT in 10–15% of the cases [2]. The incidence level of GTD changes between different countries with the highest rates in some Asian regions, the Middle East, and Africa, and it is between 0.57 and 2 per 1000 pregnancies [3], approximately 15–20% of complete moles and 0.5–5% of partial moles transform into malignant forms [4]. The established risk factors for a complete mole are pregnancy at extremes of maternal age (<21 or>40 years old), and prior molar pregnancy, which increases the risk to 10 times for sporadic complete moles. The most common type of GTD is the benign hydatidiform mole, complete or partial, which is characterised by excessive trophoblastic proliferation and abnormal embryonic development, and it could be sporadic or recurrent [5].

Ultrasound is the standard imaging modality for identifying molar pregnancy. Classically, a ‘snowstorm pattern' has been described, resulting from the presence of a complex vesicular intrauterine mass containing many ‘grape-like' cysts with no foetal tissue (CHM) or only partial tissue (PHM). Ultrasound evaluation of the adnexa can also reveal theca lutein cysts, due to ovarian stimulation by abnormally elevated beta-human chorionic gonadotropin (hCG) levels. Moreover, other different imaging techniques in the diagnosis and management of GTD, such as pelvic magnetic resonance imaging can represent a problem-solving tool to assess the depth of myometrial invasion and extrauterine disease spread in equivocal and complicated cases [6].

CHM histology consists of hydropic villi in semitransparent vesicles of variable size with the absence of normal placenta tissue, and in some cases of early CHM, no clear evidence of abnormal villi could be detected. Otherwise, in the case of PHM, normal cytotrophoblast tissue or foetal/adnexal part could be detected. The cytogenetics of CHM and PHM are different: typically, CHM is diploid and has 46 XX chromosomes with both X chromosomes from the paternal line, whereas PHM is triploid with the maternal and paternal genetic origin [2]. The recurrence of two or more HM in the same patient is defined as recurrent hydatidiform moles (RHMs). RHMs may be sporadic, occurring in a single individual in a family, or may be familiar as a biparental mole that has both a maternal and a paternal contribution, due to an autosomal recessive defect in the female germ line [7]. Clinical symptoms are represented by a large spectrum of manifestations: the most common symptom is represented by first-trimester metrorrhagia, whereas hyperemesis, hyperthyroidism, and preeclampsia could be frequently associated with patients affected by GTD. In most cases, RHMs can be asymptomatic and the early and accurate diagnosis of the disease can be made thanks to both laboratory and ultrasonographic examinations, whereas in some cases only the histopathological examination may be useful in early diagnosis of GTD [8]. However, hCG measurement is the mandatory laboratory analysis, and higher blood levels with respect to physiological pregnancy, may report the suspect of the disease. We show a case report of RHMs in a woman with two previous episodes of the disease, following current guidelines about the management of a woman with consecutive molar pregnancies, focusing on a fertility approach as a future pregnancy option.

## 2. Case Report

**Figure 1:**
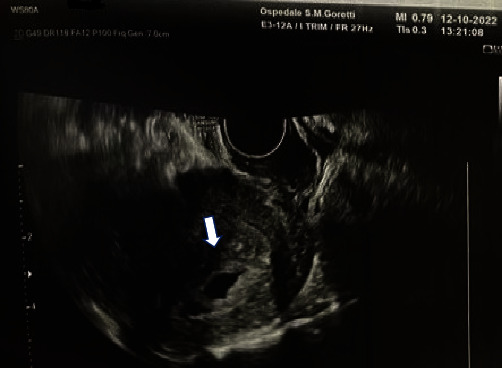
Pelvic TV ultrasound scan at 5 weeks of amenorrhea. The image shows an anechoic intrauterine scan compatible with a hemerion-free gestational chamber without cardiac activity and diffuse trophoblast characterized by an irregular profile. Both adnexa are normal. Reassessment is required after a week.

**Figure 2:**
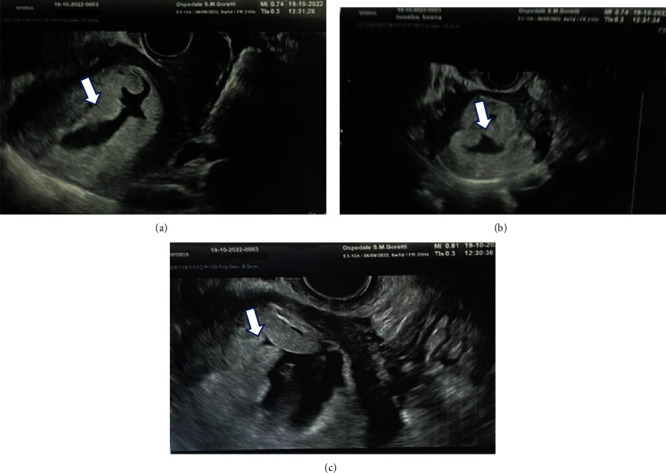
Pelvic TV ultrasound at 6 weeks of amenorrhea in longitudinal (a), transverse (b), and magnified scan (c). Ultrasound scan show a small embrionary site without cardiac activity and a diffuse trophoblast with an irregular and breasted profile in its intracavitary portion. The pictures depose for internal abortion and suspected molar degeneration.

We present a case of a 36-year-old Asian patient, gravida 3, para 0, at 5 weeks of amenorrhea, presented at our attention at the Obstetrics and Gynecology Unit, Santa Maria Goretti Hospital, Latina, in October 2022. The pregnancy was spontaneously conceived, the woman was in good global condition and was not affected by diseases or allergies. Obstetrical anamnesis of the patient reported two previous episodes of GTD, a PHM, and a CHM, interrupted at 14 and 8 weeks, respectively, and a spontaneous pregnancy loss at week 7 of amenorrhea (years 2017, 2019, and 2021). In her previous pregnancies, the patient had visited three different other hospitals and did not bring any related documentation to our attention for inspection. The patient also reports that she was adopted, therefore, we cannot trace her family history, especially relating to previous cases of repeated miscarriages or molar pregnancies in her family tree. All the previous pregnancies were conceived with the same partner. After our first transvaginal (TV) ultrasound evaluation at week 6 of amenorrhea (Figure 1 and a result of a blood hCG value of 60,000 mIU/ml, it was decided to repeat a second ultrasound evaluation after one week. A second TV ultrasound evaluation was performed on 19 October 2022, confirming the suspicion of a GTD (Figure 2). Then, we decided to hospitalize the patient and evacuate the suspected molar pregnancy. At the moment of recovery, preoperatory complete blood examinations were performed including complete blood count with platelet determination clotting, renal and liver function studies, blood type with antibody screen, and determination of hCG level, which showed a value of 185,000 mIU/ml. We also performed a preoperatory electrocardiorgram (ECG) and preevacuation chest X-ray.

**Figure 3:**
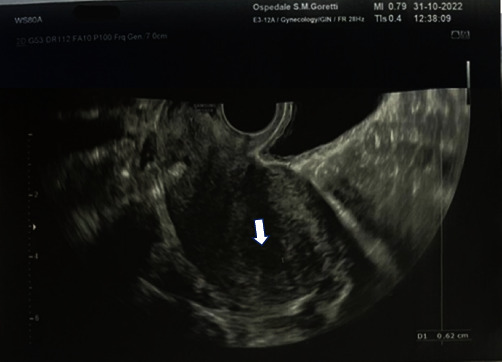
Pelvic TV ultrasound performed after the surgical procedure (D&C with suction evacuation), showing a regular thin endometrial line.

Dilatation and curettage (D&C) with suction evacuation was performed on 20 October 2022, under ultrasound guidance. The procedure was performed in general anaesthesia with a 12–14 mm suction cannula and 10 IU of intravenous oxytocin. During and after the procedure blood loss was regular, without the need for blood transfusion, as well as Rhesus immune globulin prophylaxis was not performed because of the positive blood group of the patient. The woman was discharged from the hospital on the same day of the operation in global and local good condition, after performing a TV ultrasound showing a thin and regular endometrial line (Figure 3), and with all indications of follow-up examinations. The histological examination reported hydropics microvilli, and low residues of cytotrophoblast, depending on PHM. The karyotype was performed reporting a triploid chromosomic set (69 XXY). A weekly follow-up of beta-hCG hormone was performed until negative hCG test: 202 mIU/ml on 24 October 2022, 25 mIU/ml on 8 November 2022, 8 mIU/ml on 16 November 2022, and <5 mIU/ml on 16 December 2022. Subsequently, the patient was scheduled for a monthly follow-up for six months until the return to baseline value beta-HCG. The genetic examination was not performed because of the lack of consensus of the patient due to personal reasons. A combined oral contraceptive therapy was chosen and tailored according to the clinical features and needs of the patient: estetrol 14.2 mg–drospirenone 3 mg combined oral contraceptive. The intrauterine contraceptive device was not administered, due to the high chance of uterine perforation [9]. At the same time, in vitro fertilization (IVF) was proposed, with specific oocyte donation, to avoid possible future episodes of RHMs.

## 3. Discussion

GTD is still a complex and intriguing obstetrical disease, especially in the case of RHMs, where early diagnosis is still the best practice to correctly manage and treat this kind of disease. Ultrasound is the gold standard imaging technique to make a diagnosis, where a typical honeycomb appearance of a complete mole is rarely seen, especially in the first trimester, and there is an absence of foetal parts and cystic appearance of the placenta [10]. Therefore, molar pregnancy could be detected on histologic examination after the evacuation of spontaneous abortion or a suspected molar, where only the histological examination gives a certainty diagnosis. Indeed, in women of reproductive age with abnormal bleeding or symptoms that could be caused by a neoplastic obstetrical disease, hCG levels should be evaluated to facilitate early diagnosis and treatment of GTD. In patients with molar pregnancy, the most suitable method is D&C with suction evacuation, which represents a safe and short procedure, with no difference in the incidence of subsequent GTN with respect to sharp curettage [11]. In addition, it is crucial to conduct the D&C with suction evacuation by an expert operator with a Karman cannula at least 12–14 mm in diameter, administering intravenous oxytocin after the cervix dilatation. The oxytocin administration must continue for several hours postoperatively, to enhance uterine contractility and decrease blood loss, according to the recent FIGO guidelines of 2021 [2]. After molar evacuation, all patients should be monitored with serial blood measurement of hCG, to diagnose and treat malignant sequelae promptly, according to ACOG guidelines [12].

Moreover, those patients should be encouraged to start as soon as possible with a contraceptive therapy, such as oral contraceptives, that have been proven to be safe and effective during post-treatment monitoring, reducing the incidence of post-molar GTD or altering the pattern of regression of hCG values [13].

Cases of molar pregnancy, although rare, are crucial to be early recognised and to be appropriately managed. Causes leading to a molar pregnancy underlying different etiopathogenetic pathways and complex genetic mechanisms, while clinically, patients with RHMs cannot be distinguished from non-recurrent sporadic moles, and the histological analysis is not specific in cases of RHMs. Indeed, HMs are of biparental origin, with familiar clustering of *NLRP7* or *KHDC3L* gene mutations. Recently, *PADI6* has also been identified to be responsible for RHMs [14, 15]. It is suggested that these three genes function in setting the genomic imprinting process, in specific *NLRP7* mutations have been implicated in 48–80% of RHMs cases, whereas mutations in *KHDC3L* are reported in 10–14% of these patients with no NLRP7 mutations [16]. Homozygote or compound heterozygote mutations of these three genes have been observed in most of the affected women, while there are still few fractions of RHM patients with the unidentified responsible gene. Regarding a recent study, patients with a homozygous mutation in *NLRP7* can have live birth with egg donation [17]. Indeed, the option of oocyte donation can be the best reproductive strategy, which can support mutations of those genes in patients of childbearing age [18]. Akoury et al., reported three patients affected by RHMs, with two NLRP7 defective alleles that had a total of four live births from donated oocytes, whereas patients with two defective alleles in NLRP7 may have live births from spontaneous conception from their oocytes in 1% of their pregnancies [19]. Unfortunately, in our case, we could not have the possibility to perform the cytogenetic patient assessment, due to personal and ethical patient motivation.

Furthermore, it appears that, in women who have at least two episodes of molar pregnancy, reproductive options are currently limited, while assisted reproductive technology may help to achieve normal fertilisation of oocytes [20]. A recent case report presents a case of oocyte donation performed in a patient affected by five spontaneous pregnancies with a negative outcome: a spontaneous miscarriage and four CHM, with a genetic test carried out with two heterozygous mutations in the *NLRP7* gene. The IVF technique enabled a complication-free, singleton pregnancy that resulted in a healthy term live birth female [21]. In our case, we invited our patient to embrace the hypothesis of heterologous IVF techniques, such as oocyte donation, and to perform the analysis of the DNA for sequencing and detecting specific genes involved in RHMs.

Finally, in cases of molar pregnancy, especially patients with repeated episodes, it is crucial an early diagnosis and to start appropriate treatment as soon as possible. Even though the mechanisms leading to a molar pregnancy are complex and not completely known, especially in cases of RHMs where familiar genetic clusters are involved, and no etiopathogenetic treatment is currently available, the option of oocyte donation is the best reproductive strategy, which can support this condition for a successful pregnancy.
